# Molecular Parentage Analysis Is Essential in Breeding Asian Seabass

**DOI:** 10.1371/journal.pone.0051142

**Published:** 2012-12-05

**Authors:** Peng Liu, Jun Hong Xia, Grace Lin, Fei Sun, Feng Liu, Huan Sein Lim, Hong Yan Pang, Gen Hua Yue

**Affiliations:** 1 Molecular Population Genetics Group, Temasek Life Sciences Laboratory, National University of Singapore, Singapore; 2 Marine Aquaculture Center, Agri-Food & Veterinary Authority of Singapore, Singapore; Kansas State University, United States of America

## Abstract

In aquaculture species, maintaining pedigree information and genetic variation in each generation is essential, but very difficult. In this study, we used nine microsatellites to genotype 2,520 offspring from four independent full-factorial crosses (10 males ×10 females) of Asian seabass to reconstruct pedigree and monitor the change of genetic variations. In all four crosses, over 96.8% of the offspring could be assigned to their parents, indicating the high power of the nine microsatellites for parentage assignment. This study revealed several interesting results: (1). In all four crosses, the contribution of parents to offspring was significantly uneven, and some dominant breeding fishes (i.e. brooders) were found; (2). In two mass crosses where the brooders were carefully checked for reproductive status, a majority (≥90%) of brooders contributed to offspring, whereas in another two crosses, where the brooders were randomly picked without checking reproductive status, only a few brooders (40.0–45.0%) produced offspring; (3). Females had more problems in successful spawning compared to males; and (4). In the two crosses where a few brooders produced offspring, there was a substantial loss in allelic (24.1–34.3%) and gene (20.5–25.7%) diversities in offspring, while in the other two crosses, the majority of allelic (96.8–97.0%) and gene diversities (94.8–97.1%) were maintained. These observations suggest that a routine molecular parentage analysis is required to maintain both allelic and gene diversity in breeding Asian seabass.

## Introduction

Selective breeding has led to substantial genetic improvement of some aquaculture fish species, such as salmon, rainbow trout, tilapia, carps and oysters [1]. Pedigree information is essential in estimating genetic parameters (e.g. heritability) and breeding value [2]. Unlike livestock, it is difficult to tag young fishes physically. Therefore, for breeding aquaculture species, offspring from each family must be raised separately until they are big enough to be physically tagged. This approach is not only labour and space intensive, but also introduces more environmental factors in the estimation of genetic parameters. Due to the rapid development of sequencing technologies [3], polymorphic and co-dominant DNA markers (e.g. microsatellites) have been isolated and characterized in almost all aquaculture species [4]. Genotyping offspring and parent candidates with microsatellite markers allows retrospective assignment of individuals to family groups even after different progeny groups had been reared communally after hatching. DNA markers have already been used in parentage assignment in several aquaculture species, such as Atlantic salmon [5], common carp [6], shrimp [7], [8] and Asian seabass [9], [10]. These previous studies have shown that some hatchery practices caused the reduction of genetic diversity in hatchery populations [5], [9]. In conventional breeding programs for aquaculture species, it is difficult to maintain genetic diversity for many generations, as the phenotypic selection usually selects a few elite individuals from a few families and mating of related individuals is unavoidable, which leads to inbreeding [1]. It is well known that inbreeding can result in the depression of survival and growth traits, and limit potential for continuous selection [11]. Once reliable pedigree information is available, selection can be conducted within each family, and mating can be arranged in unrelated individuals to minimize inbreeding.

The Asian seabass, *Lates calcarifer*, belongs to the family Latidae of order perciformes [12]. It is a catadromous fish species. In Australia, it is called barramundi. This species is widely distributed in the Indo-West Pacific region from the Persian Gulf, throughout Southeast Asia to northern Australia [12]. This fish has been cultured in Thailand, Malaysia, Singapore, Indonesia and Australia, and is one of the most well known marine and freshwater foodfish species in Southeast Asia. Its global annual production was 40,000 metric tons [13]. To facilitate the breeding of this species, some genomic tools have been developed since 2001, such as microsatellites [14], [15], SNPs in genes [16], a BAC and several cDNA libraries [17], [18], linkage maps, and BAC-based physical maps [19], [20], [21]. Microsatellites have been used to study genetic diversity in the wild and in hatcheries [22], to reconstruct pedigrees for estimating genetic parameters [10], and conduct linkage and QTL mapping [19], [20], [23], [24]. A previous study on parentage analysis using microsatellites revealed loss of genetic diversity due to hatchery culture practices in barramundi in Australia [9]. Since 1998, we have worked on increasing the growth rate of the Asian seabass through breeding [14]. However, little is known about fertilization success and genetic change in different generations in hatcheries of Asian seabass in Asia.

In this study, we used nine microsatellites to genotype 80 breeding fishes (i.e. brooders) and 2,520 offspring from four full-factorial mass crosses (each containing 10 males ×10 females) in our hatchery in order to analyze the contribution of parents to offspring as well as to monitor the change in genetic diversity from different crosses so as to facilitate the breeding program of Asian seabass. Our data suggests that careful selection of brooders for spawning and routine molecular parentage analysis is critical to maintain both allelic and gene diversity for breeding Asian seabass.

## Materials and Methods

### 1. Ethics statement

All handling of fishes was conducted in accordance with the guidelines on the care and use of animals for scientific purposes set up by the Institutional Animal Care and Use Committee (IACUC) of the Temasek Life Sciences Laboratory, Singapore. The IACUC has specially approved this study within the project “To ensure self-sufficiency of safe seafood for Singapore through the development of aquaculture genomic tools for marker-assisted selective breeding of tropical marine foodfish” (approval number is TLL (F)-07-009).

### 2. Breeding fishes and offspring

Forty male and 40 female breeding fishes (i.e. brooders) selected from an existing broodstock containing over 500 adult (3–4 years old) *L. calcarifer* individuals were used in this study. The 80 brooders were sexually matured and raised in 60-ton tanks located in the Marine Aquaculture Centre (MAC), Singapore. These fishes were evenly divided into four groups. In the first two groups, both males and females were carefully checked for readiness for spawning by cannulation (e.g. inserting a cannula into the reproductive tract to check the readiness of reproduction) as described [25]. In the other two groups, the brooders were used for spawning without prior checking of their reproductive status. Ten males, together with ten females, from each group were induced hormonally during their natural spawning rhythm, and placed in a 20-ton tank for a mass-spawning. Fin clips of the 80 brooders were collected and kept in absolute ethanol for later DNA extraction. Eggs from the four crosses were collected on the second night of spawning, and were brought to two one-ton incubation tanks located in the MAC for hatching. Over 1,000 larvae were collected from each cross after hatching.

### 3. DNA extraction

Total DNA from each fish was extracted using a method developed by us [26]. Extracted DNA was eluted in distilled water. DNA quality and quantity were examined using electrophoresis on 1.0% agarose gels and Nanodrop (Thermo Scientific), respectively.

### 4. Genotyping of microsatellites

To reconstruct pedigrees, all offspring from the four mass crosses, and their parents were genotyped with nine microsatellites (*Lca008*, *Lca020*, *Lca021*, *Lca058*, *Lca064*, *Lca069*, *Lca070*, *Lca074* and *Lca098*) developed previously [14], [15]. Multiplex PCR for all nine loci was conducted on PTC-100 PCR machines (MJ-Research) to amplify each microsatellite as described previously [27]. Briefly, the following program was used: 94°C for 2 min., followed by 35 cycles of denaturing at 94°C for 30 s, annealing at 55°C for 30 s, and extension at 72°C for 30 s, then a final step of 72°C for 5 min. Each 25 µl PCR reaction consisted of 1× PCR buffer (Finnzymes) with 1.5 mM MgCl_2_, 50–400 nM of each PCR primer of the nine markers, 50 µM of each dNTP, 40 ng genomic DNA and one unit of DNA-polymerase (Finnzymes). Products were analyzed using an ABI3730xl DNA sequencer (Applied Biosystems). Fragment sizes were determined against the size standard ROX-500 (Applied Biosystems) using the GeneMapper version 4.1 (Applied Biosystems).

### 5. Data analysis

The genotypes of parents and offspring were used to carry out the parentage assignment to reconstruct pedigrees using software PAPA v2.0 [28]. The assignment was based on a maximum-likelihood of each potential parental pair assuming basic hypotheses such as that reproduction was sexual, with random mating within each cross, and that all potential parents produced offspring equally. Assignment procedures were performed with the following parameters: known sex of parents and no genotyping errors, thus maintaining the absolute certainty of true allele transmission from parents to their offspring.

Allele number, allele frequency, expected and observed heterozygosity, and inbreeding index (*f*) in parents and offspring were calculated using the program: Genetic Data Analysis (GDA) [29]. The number of offspring contributed by each individual brooder was then determined and used to calculate its percent contribution to the total cohort. Chi-square (*X* ^2^) analyses were used to determine if contribution levels were significantly different between brooders.

Effective population size (Ne) was calculated according to the formula Ne = 4 (N−2)/((Ks+Vs/Ks)+(Kd+Vd/Kd)−2) [6], where N is the number of offspring sampled, Ks and Kd are the mean number of offspring per sire and per dam, and Vs and Vd are the variances of sire and dam family sizes. In this equation, the differences in sample size and the differential contribution levels made by individual broodstock to the resulting cohort of progeny were taken into account.

## Results and Discussion

### 1. Power of the nine microsatellites in parentage assignment

810, 570, 570 and 570 offspring from the mass crosses 1, 2, 3, and 4 were genotyped with nine microsatellites, respectively. In mass cross 1, all 810 offspring were successfully genotyped. Among them, 784 (96.8%) individuals were assigned to specific parent pairs. In mass crosses 2, 3 and 4, 562, 570 and 570 offspring, respectively were successfully scored. Among them, 554 (98.6%), 566 (99.2%), and 563 (98.8%) in crosses 2, 3 and 4 were assigned to specific parent pairs, respectively. The overall correct assignment rate (>96.8%) in this study is similar to the success rate in our previous study using the same set of microsatellites [10]. These data suggest the high power of the nine microsatellites in parentage assignment of Asian seabass.

### 2. Contribution of parents to offspring in four full-factorial crosses

In this study, the parentage assignment in four mass crosses revealed several interesting results. Firstly, in all four mass crosses, some brooders did not contribute offspring. In mass cross 1, all 10 male brooders and 9 of 10 females produced offspring, and generated 51 families (Table 1). In mass cross 2, nine of the 10 males and nine of 10 females generated progeny in 40 families (Table 1). In mass cross 3, only five male and two female brooders produced offspring and generated only five families (Table 1). In mass cross 4, six male and three female brooders produced offspring and generated eight families (Table 1). The brooders that did not contribute offspring, especially those in cross 3 and 4, could have been in a poor physiological condition and therefore, did not contribute at all. It is also possible that they did not reproduce because of their inability to spawn or produce good-quality gametes. These data, together with those published by others [9], indicate that in order to ensure that most brooders produce offspring, it is essential to check the reproductive status before putting brooders for mass spawning.

**Table 1:** Number of offspring in each family of four mass crosses of Asian seabass.

Cross 1	Male											
Female	1	2	3	4	5	6	7	8	9	10	total	%
**1**	**1**	0	**1**	0	7	0	0	0	0	**4**	**13**	**1.7**
**2**	0	0	**6**	**1**	**6**	0	0	**8**	0	0	**21**	**2.7**
**3**	0	**52**	**155**	**23**	**6**	**36**	**3**	0	0	**5**	**280**	**35.7**
**4**	0	**104**	**11**	**1**	**1**	**2**	0	**2**	0	0	**121**	**15.4**
**5**	**14**	**3**	**2**	0	**42**	**8**	0	**3**	**28**	**10**	**110**	**14.0**
**6**	**37**	**14**	0	**4**	**1**	**34**	0	0	**1**	**15**	**106**	**13.5**
**7**	0	0	0	0	0	0	0	0	0	0	**0**	**0**
**8**	0	0	0	0	**1**	**1**	0	0	0	0	**2**	**0.3**
**9**	**1**	0	0	**1**	0	**1**	0	0	0	**1**	**4**	**0.5**
**10**	**2**	**64**	**3**	**20**	**4**	**10**	**1**	**16**	**1**	**6**	**127**	**16.2**
**Total**	**55**	**237**	**178**	**50**	**68**	**92**	**4**	**29**	**30**	**41**	**784**	-
**%**	**7.0**	**30.2**	**22.7**	**6.4**	**8.7**	**11.7**	**0.5**	**3.7**	**3.8**	**5.2**	-	**100**
**Cross 2**	**1**	**2**	**3**	**4**	**5**	**6**	**7**	**8**	**9**	**10**	**total**	**%**
**1**	0	0	0	0	0	0	0	0	0	0	**0**	**0**
**2**	0	**16**	0	**14**	**9**	**29**	0	**5**	**3**	**2**	**78**	**14.1**
**3**	0	0	**8**	0	0	0	0	**7**	0	**3**	**18**	**3.3**
**4**	0	**8**	**4**	0	**3**	0	**19**	**1**	0	**1**	**36**	**6.5**
**5**	0	**1**	**3**	**37**	0	**2**	0	**3**	0	0	**46**	**8.3**
**6**	0	0	**2**	**10**	**24**	0	0	0	0	0	**36**	**6.5**
**7**	0	**6**	**28**	**2**	**5**	**26**	**1**	0	0	0	**68**	**12.3**
**8**	0	0	0	**5**	0	0	**22**	0	**3**	**112**	**142**	**25.6**
**9**	0	**6**	0	**10**	0	0	0	0	0	0	**16**	**2.9**
**10**	0	**38**	**4**	**16**	0	**1**	**1**	**54**	0	0	**114**	**20.6**
**Total**	**0**	**75**	**49**	**94**	**41**	**58**	**43**	**70**	**6**	**118**	**554**	-
**%**	**0**	**13.5**	**8.8**	**17.0**	**7.4**	**10.5**	**7.8**	**12.6**	**1.1**	**21.3**	-	**100**
**Cross 3**	**1**	**2**	**3**	**4**	**5**	**6**	**7**	**8**	**9**	**10**	**total**	**%**
**1**	0	0	**2**	0	0	0	0	0	0	0	**0**	**0.3**
**2**	0	0	0	0	0	0	0	0	0	0	**0**	**0**
**3**	0	0	0	0	0	0	0	0	0	0	**0**	**0**
**4**	0	0	0	0	0	0	0	0	0	0	**0**	**0**
**5**	0	0	0	0	0	0	0	0	0	0	**0**	**0**
**6**	0	0	0	0	0	0	0	0	0	0	**0**	**0**
**7**	0	0	0	0	0	0	0	0	0	0	**0**	**0**
**8**	0	**429**	**130**	0	0	**1**	0	0	**1**	**3**	**564**	**99.7**
**9**	0	0	0	0	0	0	0	0	0	0	**0**	**0**
**10**	0	0	0	0	0	0	0	0	0	0	**0**	**0**
**Total**	**0**	**429**	**132**	**0**	**0**	**1**	**0**	**0**	**1**	**3**	**566**	-
**%**	**0**	**75.9**	**23.1**	**0**	**0**	**0.2**	**0**	**0**	**0.2**	**0.5**	-	**100**
**Cross 4**	**1**	**2**	**3**	**4**	**5**	**6**	**7**	**8**	**9**	**10**	**Total**	**%**
**1**	0	0	0	0	0	0	0	0	0	0	**0**	**0**
**2**	**90**	**2**	**123**	**126**	**205**	**0**	**0**	**14**	**0**	**0**	**560**	**99.4**
**3**	0	0	0	0	0	0	0	0	0	0	**0**	**0**
**4**	0	0	0	0	0	0	0	0	0	0	**0**	**0**
**5**	0	0	0	0	0	0	0	0	0	0	**2**	**0**
**6**	**2**	0	0	0	0	0	0	0	0	0	**0**	**0.4**
**7**	0	0	0	0	**1**	0	0	0	0	0	**2**	**0.2**
**8**	0	0	0	0	0	0	0	0	0	0	**0**	**0**
**9**	0	0	0	0	0	0	0	0	0	0	**0**	**0**
**10**	0	0	0	0	0	0	0	0	0	0	**0**	**0**
**Total**	**92**	**2**	**123**	**126**	**206**	**0**	**0**	**14**	**0**	**0**	**563**	-
**%**	**16.3**	**0.4**	**21.9**	**22.4**	**36.6**	**0**	**0**	**2.5**	**0**	**0**	-	**100**

Secondly, in each of the four crosses, several dominant brooders were found, and the contribution of brooders to offspring was substantially uneven. In mass cross 1, the contribution of males (*X* ^2^ = 618, *d.f.* = 9, *P*<0.0001) and females (*X* ^2^ = 92, *d.f.* = 9, *P*<0.0001) was uneven, however, some dominant males (i.e. male 2 and 3) and females (e.g. female 3) were detected in this cross. Males 2 and 3 contributed 30.2% and 22.7% of the total offspring, while the dominant female 3 contributed 35.7% of the total offspring. In mass cross 2, sire contributions to the cohort were different (*X* ^2^ = 291, *d.f*. = 9, *P*<0.0001), with similar results from the dam contributions (*X* ^2^ = 341, *d.f.* = 9, *P*<0.0001). Males 4 and 10 fertilized eggs from four and seven females respectively, contributing to 17.0% and 21.3% of the offspring cohort. Females 8 and 10 mated with four and six males respectively, and contributed 25.6% and 20.6% of all offspring. The contribution of brooders to offspring in mass cross 3 was substantially uneven amongst the 10 males (*X* ^2^ = 2989, *d.f.* = 9, *P*<0.0001) and 10 females (*X* ^2^ = 5054, *d.f.* = 9, *P*<0.0001). The female 8 mated with five male brooders and produced 99.7% of the total offspring. The pair (male 2 and female 8) contributed 75.9% of the total offspring cohort. 99.5% of the offspring in mass cross 4 were produced by female 2 via mating with six male brooders. The contribution of female and males brooders to the offspring in this cross was substantially biased (for females *X* ^2^ = 5007, *d.f.* = 9, *P*<0.0001, and for males *X* ^2^ = 8594, *d.f.* = 9, *P*<0.0001). The pair (female 2 and male 5) produced 36.6% of all offspring. Similar uneven contribution of brooders to offspring has been found in common carp [6], salmon [5] and Asian seabass [9]. It seems that in mass crosses of aquaculture species, uneven contribution of brooders to offspring is a common phenomenon. There are a few factors that may contribute to this difference. The first is mating behaviour and mating selection of brooders [30]. The different contribution of brooders may be a consequence of sperm competition [31], resulting from variation in sperm quality. Where sperms from several males are shed into a pool of eggs, sperm competition may occur. Given the variability in reproductive success seen among brooders in this study, further investigation into whether sperm competition and mating selection play a major role in the different reproductive success of different brooders are warranted.

Thirdly, it seems that female Asian seabass encounter more spawning problems than males when it comes to successful spawning compared to males in Asian seabass as seen in mass crosses 3 and 4, where only 2–3 females spawned and over 98.0% of the offspring were from one dominant female, whereas more males were able to produce offspring. This result contrasts with that of Frost et al. [9], who reported that fertility of females was not a major limitation to spawning, as females respond well to hormone induction. The reasons for the low percentage of contribution of female brooders to offspring were not clear in our study, and warrant further study. It is also of interest to see whether the contributions of brooders to offspring could be even, if the dominant male and female brooders are removed from the mass spawning.

### 3. Changes of allelic, gene diversity and effective population size in parents to offspring in four mass crosses

**Figure 1:**
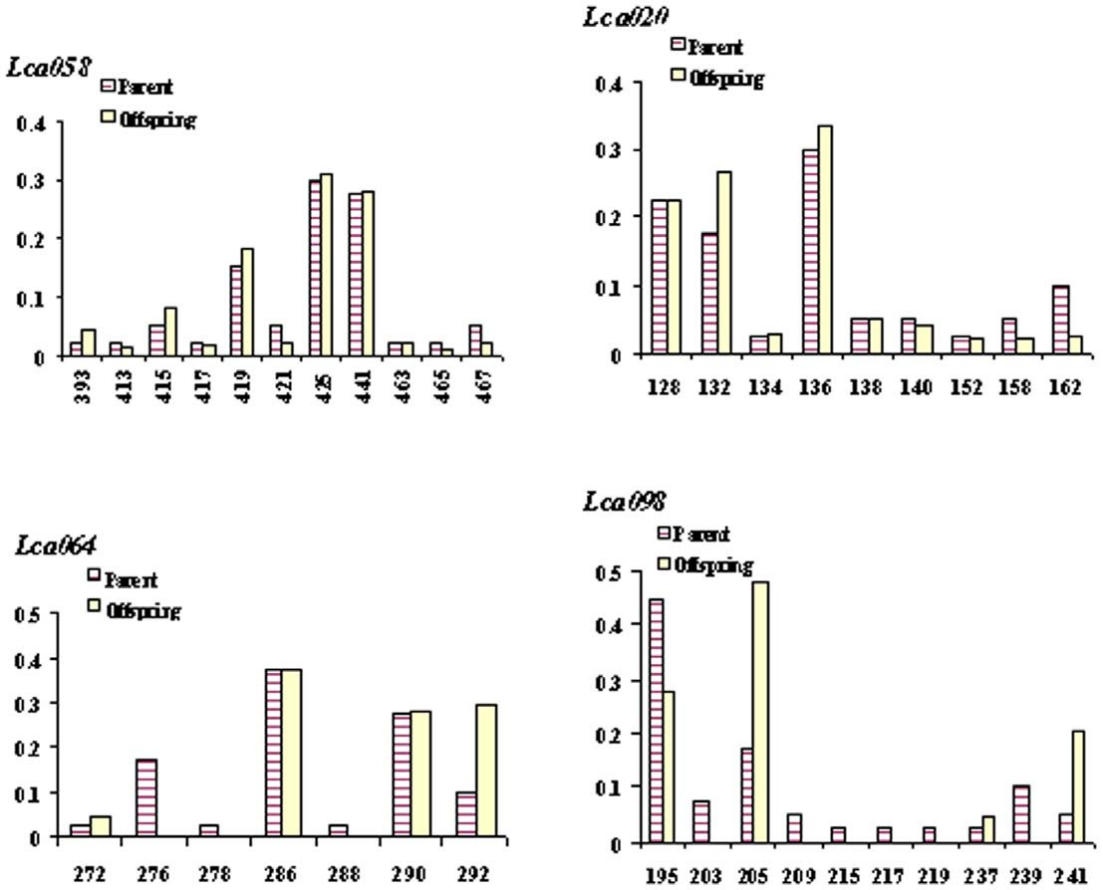
Allele frequencies of parents and offspring at four microsatellite loci in four mass crosses of Asian seabass. *Lca058* in mass cross 1; *Lca020* in mass cross 2; *Lca064* in mass cross 3 and *Lca098* in mass cross 4.

Hatchery production is critically important in commercial production and breeding for genetic improvement of traits of interest [32]. It is well known that to ensure long-term sustainability of a breeding program, it is essential to maintain high genetic diversity from generation to generation [1]. In mass cross 1 of this study, where the largest majority (95.0%) of brooders contributed to offspring, the average allele number at the nine microsatellite loci was only slightly reduced (from *A* = 6.0 in parents to *A* = 5.8 in offspring, see Table 2). Most alleles were maintained in the offspring at the nine loci (see example at the locus *Lca058* in Figure 1), whereas allele frequency changed in parents and offspring. Gene diversity (i.e. *H*e) was almost maintained in offspring (*H*e = 0.66 in offspring vs. *H*e = 0.68 in parents). The effective population size (*N*e) was 10.1 in offspring, which is much smaller than the census population size (*N* = 20) of parents, and may be the consequence of the uneven contribution of brooders. In mass cross 2, 90.0% of brooders produced offspring. The average allele number at the nine loci was 7.22 in 20 parents, whereas the number was slightly lower (7.0) in offspring (Table 2). The majority of alleles were retained in the offspring (see example at the locus *Lca020* in Figure 1). The gene diversity in the offspring was also slightly lower than that in parents (*H*e: 0.72 in offspring vs. 0.76 in parents). The effective population size was smaller in the offspring (*N*e = 13.6) than the census population size (*N* = 20) in parents. In mass crosses 3 and 4, where only a few parents produced offspring, the average allele number was substantially reduced (5.2 in offspring vs. 6.9 in parents in cross 3, and 4.4 in offspring vs. 6.7 in parents in cross 4). Some alleles with low frequency were lost in offspring. For example, in cross 3, at locus *Lca064*, three alleles (i.e. 276, 278 and 288) were lost in the offspring (see Figure 1). Similarly, in mass cross 4, at the locus *Lca098*, five alleles (203, 209, 215, 217 and 219) disappeared in the offspring (see Figure 1). The effective population size in cross 3 (*N*e = 3.2) and 4 (*N*e = 3.1) was much smaller compared to the census population size (*N* = 20) in parents. Significant reduction in genetic variation in breeding stocks in different generations has been reported in previous studies [9], [10]. These differences could be a result of the selection of a few elite candidates from a few families. In this study, we found that in mass crosses 1 and 2, where a majority of brooders contributed to the offspring, the allelic and gene diversity was almost fully conserved. However, owing to the substantial uneven contribution of brooders to offspring, the effective population size in offspring was smaller than the census size of the brooders. Thus, the even contribution of brooders to offspring could be an important issue in sustainable breeding of Asian seabass. In mass crosses 3 and 4, only fewer 50% of brooders produced offspring, and the allelic and gene diversity was substantially reduced. Furthermore, the effective population size was only 15% of the census size of the brooders. Similar substantial reduction of alleles, gene diversity and effective populations was seen in barramundi in Australia [9], salmon [5] and many other aquaculture species [33].

**Table 2:** Genetic diversity in parents and offspring in four mass crosses of Asian seabass.

	Parent					Offspring				
	*N*	*A*	*H_O_*	*H_E_*	*f*	*N*	*A*	*H_O_*	*H_E_*	*f*
Cross 1	20	6.0	0.71	0.68	−0.05	784	5.8	0.73	0.66	−0.10
Cross 2	20	7.2	0.75	0.76	0.01	554	7.0	0.77	0.72	−0.07
Cross 3	20	6.9	0.74	0.73	−0.02	566	5.2	0.66	0.58	−0.15
Cross 4	20	6. 7	0.77	0.74	−0.04	563	4.4	0.64	0.55	−0.18

*N*: number of individuals; *A*: average number of alleles at nine microsatellite loci. *Ho*: average observed heterozygosity; *He*: average expected heterozygosity, and *f*: average fixation index.

Our results and others suggest that in order to maintain the genetic variation within a breeding population, it is essential to ensure that all the brooders produce offspring. Even if all brooders contribute to offspring, the differential contribution of brooders will still cause a reduction in the effective population sizes in subsequent generations. Fortunately, in Asian seabass, artificial insemination technologies using cryopreserved sperm are available [34] to address this issue. Artificial fertilization of mixed eggs from different female brooders with mixed sperm from several male brooders may make the contribution of brooders to offspring equal. However, this remains to be tested by experiments. Parentage analysis of results from artificial insemination using microsatellites could help answer this question, and contribute to the understanding of genetic variation from generation to generation.

In conclusion, parentage analysis using nine microsatellites revealed substantially differential contribution of brooders to offspring in four mass crosses of Asian seabass. Zero or uneven contribution of brooders to offspring led to the reduction of effective population size. Female Asian seabass encountered more spawning problems than males. Artificial insemination [34] may be a way to solve the problem of uneven contribution. Our study highlights the requirement of a careful check of the reproductive status of brooders prior to spawning, and the necessity of routine parentage analysis of each mass cross using microsatellites in monitoring the change of genetic variation for breeding Asian seabass.
